# The effect of Danshen extract on lipoprotein-associated phospholipase A_2_ levels in patients with stable angina pectoris: study protocol for a randomized controlled trial - the DOLPHIN study

**DOI:** 10.1186/s13063-017-2336-2

**Published:** 2017-12-20

**Authors:** A-Di Chen, Chun-Ling Wang, Yang Qin, Liang Tian, Li-Bin Chen, Xiao-Ming Yuan, Lin-Xiu Ma, Yu-Feng Wang, Ji-Rong Sun, Hao-Sen Wang, Neng Dai, DOLPHIN investigator group

**Affiliations:** 1 Cardiology Department, Taizhou Fourth People’s Hospital, Taizhou, Jiangsu Province 225300 China; 2 Department of Science and Education, Taizhou Fourth People’s Hospital, Taizhou, Jiangsu Province 225300 China; 3 Cardiology Department, Tenth People’s Hospital of Tongji University, Shanghai, 200072 China; 4 Cardiology Department, Tenth People’s Hospital, Tongji University School of Medicine, 301 Middle Yanchang Road, Shanghai, 200072 China

**Keywords:** Chinese medicine, Angina pectoris, Danshen injection

## Abstract

**Background:**

Lipoprotein-associated phospholipase A_2_ (Lp-PLA_2_), a biomarker of oxidation and inflammation, has been associated with increased coronary artery disease risk. To date, very few studies have examined the Chinese herbal drug Danshen or its extract on Lp-PLA_2_ in patients with stable angina pectoris. In this study, we aim to investigate the effect of Danshen extract on Lp-PLA_2_ level in patients with stable angina.

**Methods/design:**

This is a randomized, single-blind, placebo-controlled, adaptive clinical trial. A total of 156 patients meeting the eligibility criteria will be randomly assigned to either the Danshen extract (DanshenDuofensuanyan injection and Danshen drop spill) group or the placebo group in a 1:1 ratio. Participants will then undergo treatment with DanshenDuofensuanyan injection or placebo (glucose) during hospitalization, followed by open-label Danshen drop spill (30 pills/day) in Danshen extract group for 60 days after discharge. Because this is an adaptive trial, two interim analyses are prospectively planned. These will be performed after one-third and two-thirds of the patients, respectively, have completed the trial. On the basis of the results of these interim analyses, a data monitoring committee will determine how to modify aspects of the study without undermining the validity and integrity of the trial. The primary outcome measure is the serum level of Lp-PLA_2_ in the Danshen extract group and the placebo group. The secondary outcomes include the proportion of patients who show a clinically significant change, which is defined as at least a 20-point improvement in angina frequency score on the Seattle Angina Questionnaire and the carotid intima-media thickness, which will be measured using ultrasound. Other secondary efficacy and safety outcomes will also be assessed.

**Discussion:**

This study will provide evidence that Danshen extract is beneficial for stable angina and may establish a possible mechanism of Danshen treatment effects on cardiovascular disease. This study may also validate an objective blood test (LP-PLA_2_ level) for assessing the effectiveness of Danshen therapy in patients with stable angina pectoris.

**Trial registration:**

ClinicalTrials.gov, NCT02870764. Registered on 13 August 2016.

**Electronic supplementary material:**

The online version of this article (doi:10.1186/s13063-017-2336-2) contains supplementary material, which is available to authorized users.

## Background

Cardiovascular disease is the leading cause of death worldwide, accounting for 17.3 million deaths per year, and is predicted to reach 23.6 million annually by 2030 [1]. Chronic stable angina makes up 50% of all patients with coronary artery disease (CAD) [2]. Its symptoms are highly related to the development of atherosclerotic plaque that obstructs at least one large epicardial coronary artery and triggers an imbalance between myocardial oxygen supply and demand.

Inflammation has been found to play critical and continuous roles on the initiation and progression of atherosclerosis and CAD [3, 4]. Of all the key inflammation biomarkers, lipoprotein-associated phospholipase A_2_ (Lp-PLA_2_) is an enzyme secreted predominantly from atherosclerotic plaques by macrophages and neutrophils and then circulates in the bloodstream [5]. This offers several advantages relative to other inflammatory markers, including specificity for vascular inflammation, minimal biovariability, and stability in cases of myocardial ischemia. Several studies have demonstrated that Lp-PLA_2_ is strongly expressed in macrophages in the fibrous cap of coronary lesions prone to rupture as well as within the necrotic core [6, 7]. Furthermore, the intensity of Lp-PLA_2_ staining is closely related to plaque vulnerability [7]. In a prospective cohort study of patients undergoing carotid endarterectomy, Lp-PLA_2_ expression was higher in plaques from patients with CAD than in plaques from those without CAD [8]. In addition, Lp-PLA_2_ mass was demonstrated to predict future cardiovascular events in patients with stable CAD [9–12]. In a majority of studies, Lp-PLA_2_ has been shown to be an independent predictor of future events, even after adjustment for several conventional risk factors. All these findings strongly suggest that decreasing the Lp-PLA_2_ level in patients with CAD may improve their clinical outcomes and that the serum Lp-PLA_2_ level may be used as a marker for the effectiveness of treatments for CAD.

Given Lp-PLA_2_’s role as a key modulator of oxidative stress, inflammation, and atherosclerosis [13] and that it overlaps with the effects of Danshen therapy [14], Danshen likely works through lowering Lp-PLA_2_ levels to achieve its efficacy in inhibiting oxidative stress and inflammation. Danshen, the dried root of *Salvia miltiorrhiza*, is one of the most versatile Chinese herbal drugs. Danshen can improve microcirculation, vasodilate coronary vessels, suppress inflammation, and protect against myocardial ischemia [14–16]. It is widely used either alone or in combination with other herbal ingredients for patients with CAD and other cardiovascular diseases in China and, to a lesser extent, in other countries, including the United States.

Authors of two previous systematic reviews concluded that Danshen extract added to conventional therapy in patients with CAD reduced major adverse cardiac events and improved patient survival [17, 18]. However, the mechanisms behind these findings have not been studied before. We hypothesized that Danshen treatment acts on Lp-PLA_2_ to beneficially affect patients’ clinical outcomes. Thus, the aim of our present trial is to investigate the effect of Danshen extract on serum Lp-PLA_2_ levels in patients with stable angina.

## Methods/design

### Design

The Effect of Dan-shen Extract on Lipoprotein-associated PHospholipase A2 Levels in Patients with Stable Angina Pectoris study (DOLPHIN study) is a prospective, randomized, single-blind, placebo-controlled study designed to evaluate the effect of Danshen extract (DanshenDuofensuanyan, also known as *salvianolate*) and Danshen drop pills, with both these two drugs having as their main active ingredient Danshen extract (*Salvia miltiorrhiza*), on Lp-PLA_2_ levels in patients with stable angina. The trial is registered with ClinicalTrials.gov (NCT02870764), and the full trial protocol can be accessed at https://clinicaltrials.gov/ct2/show/NCT02870764?term=dolphin&rank=4.

### Patients

**Fig. 1:**
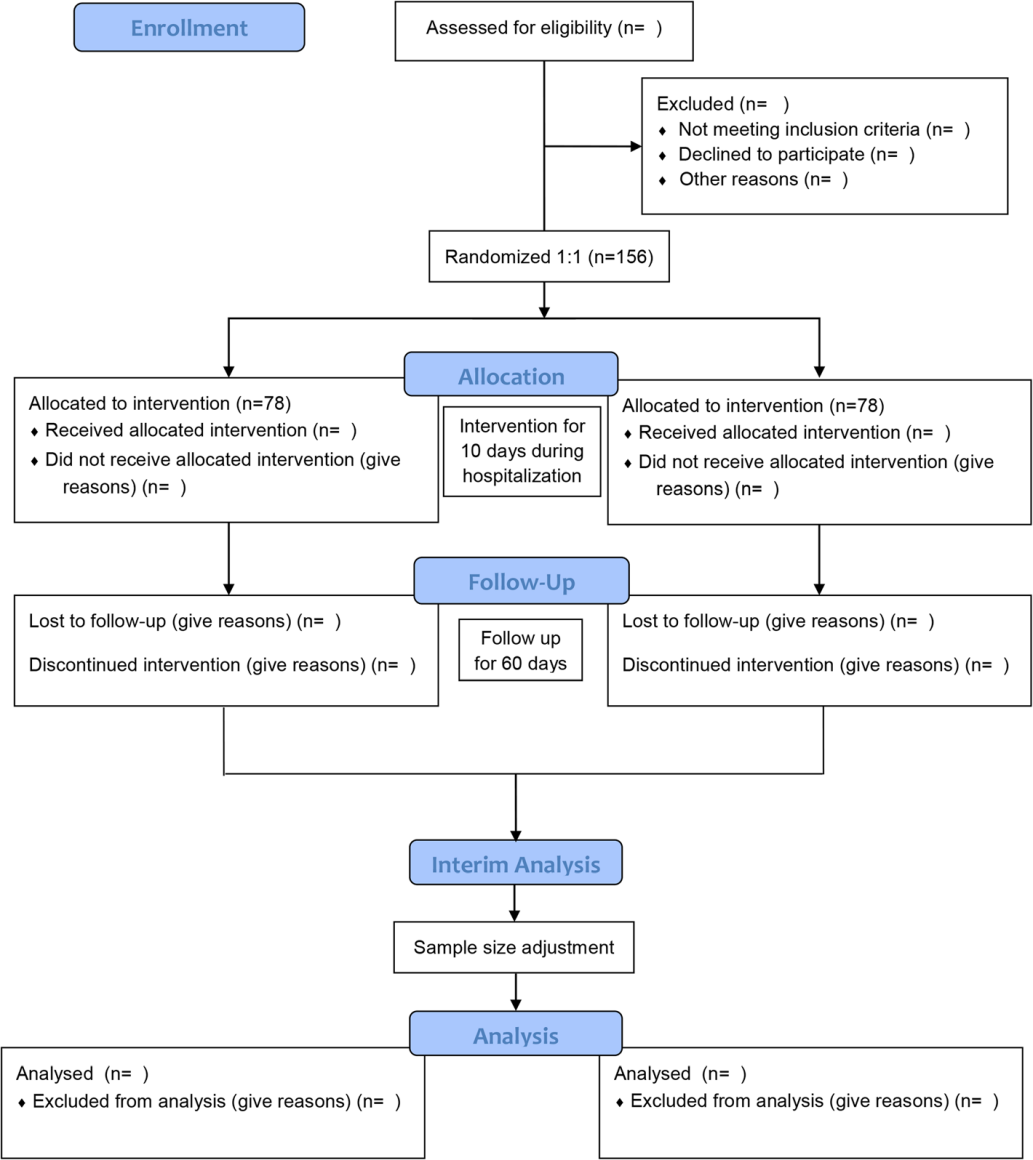
Flowchart of the trial

**Fig. 2:**
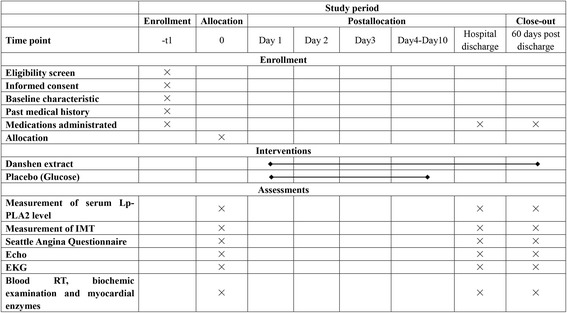
The schedule of enrollment, interventions, and assessments. *Blood RT* Blood routine test, *Echo* Echocardiography, *EKG* Electrocardiography, *IMT* Carotid intima-media thickness, *Lp-PLA*_*2*_ Lipoprotein-associated phospholipase A_2_

The DOLPHIN study will be performed in Taizhou Fourth People’s Hospital, Taizhou, China. Patients with stable angina will be recruited. The study flowchart is shown in Fig. 1, and detailed inclusion and exclusion criteria are listed in Table 1. The detailed study schedule is provided in Fig. 2, and the Standard Protocol Items: Recommendations for Interventional Trials (SPIRIT) checklist [19] is provided in Additional file 1.

**Table 1:** Inclusion and exclusion criteria

Inclusion criteria	Exclusion criteria
1. Aged 18–75 years 2. Written informed consent provided 3. Patients with a clinical diagnosis of chronic stable angina who fulfill one of the following conditions: a. Symptoms that support the diagnosis of chronic angina, and/or a history of an abnormal exercise response limited by angina, and/or electrocardiographic changes b. A history of myocardial infarction and ST-T changes c. Stenosis greater than 50% in at least one major epicardial coronary artery, as shown by coronary angiography or computed tomographic angiography d. Coronary heart disease confirmed by radionuclide angiocardiography 4. Patients with moderate angina pectoris, which is defined as grade II or III on the Canadian Cardiovascular Society Angina Grading Scale	1. Patients with severe complications that would complicate the condition, as assessed by the investigator, including liver or renal dysfunction, severe cardiopulmonary dysfunction, pulmonary hypertension, chronic obstructive pulmonary disease, a history of epilepsy, or cerebral hemorrhage 2. Patients who experienced myocardial infarction or who were classified as grade IV on the Canadian Cardiovascular Society Angina Grading Scale within the preceding 3 months 3. Patients with chest pain that is caused by any other disease (e.g., acute myocardial infarction, severe neurosis, menopausal syndrome, or hyperthyroidism) 4. Patients with a history of drug-induced bleeding or a history of bleeding caused by warfarin 5. Patients with a history of hematopoietic disorder 6. Patients who have had surgery within the previous 4 weeks or who have a hemorrhagic tendency 7. Women who are pregnant or lactating or who have a positive pregnancy test, or women who have a menstrual period at baseline 8. Patients who are participating in other trials or who have participated in other trials within the past 3 months 9. Patients with a history of allergy or with a known or suspected allergy to the study drug 10. Patients with a known or suspected history of alcohol or drug abuse within the past 2 years 11. Patients with a mental disorder 12. Family members or relatives of the study center staff 13. Inability to adhere to study procedures

### Statistical hypotheses and sample size determination

According to previous studies [20–22], Lp-PLA_2_ level was reduced by around 30% in the medication group but by only 10% in placebo group. It is hypothesized that a reduction of more than 30% is clinically significant for the Danshen extract group. Therefore, the number of subjects required is initially estimated to be at least 124 (one-sided test, α = 0.05, β = 0.2) [23]. To allow for a 20% dropout rate, a total of 156 patients will therefore be recruited. Patients will be randomized into the Danshen extract group or the control (glucose) group in a ratio of 1:1. According to the adaptive design principle, we will have two interim analyses, which will be performed when one-third (*n* = 52) and two-thirds (*n* = 52104) of the patients have completed the trial, and the sample size will be adjusted accordingly.

### Randomization and blinding

Randomization will be carried out by an independent randomization coordinator from Taizhou Fourth People’s Hospital according to a 1:1 intervention-to-control ratio. The physicians, patients, evaluators, and statistician will be blinded to which treatment group the participant has been allocated, except for nurses who will deliver the drugs, and the nurses who deliver the drugs will be blinded to the physicians, evaluators, and statistician and will not participate in the data collection or analysis.

Participants and relevant researchers will be blinded to DanshenDuofensuanyan (salvianolate) injection therapy or placebo treatment group assignments during the whole study period. Because the color of DanshenDuofensuanyan injection and 5% glucose injection are different, the dropping bottles will be wrapped in sealed, shaded bags during infusion, and brown infusion devices will be used for infusion. The nurses will check and verify the integrity of wrapping throughout the infusion.

### Treatment

According to the Chinese guidelines for the diagnosis and treatment of chronic stable angina [24], participants included in this trial will receive the standard conventional therapy as a basic treatment, including oral nitrates, statins, β-blockers, and antiplatelet agents, in addition to the Danshen extract treatment or placebo treatment. Because statins can have an effect of reducing Lp-PLA_2_ levels in a dose-dependent manner, all participants will receive atorvastatin 20 mg per night. If the patients are not suitable for receiving atorvastatin therapy, another isodose statin will be used. All treatments will be recorded in detail in the patients’ medical records as well as in their case report forms (CRFs).

Participants in the Danshen extract group will be treated using DanshenDuofensuanyan (200 mg per day) plus 0.9% saline injection (250 ml intravenously per day), whereas participants in the placebo group will be treated using placebo (glucose 200 mg per day) plus 0.9% saline injection (250 ml intravenously per day). If the patient has diabetes mellitus, glucose will be balanced with insulin. All of the included patients will undergo a 70-day treatment regimen during which patients in the Danshen extract group will receive a 10-day of course DanshenDuofensuanyan therapy in a double-blind manner and then continue to receive Danshen drop pills (a Danshen extract formulated for oral administration) unblinded, whereas the patients in the control group will receive standard medical care only. Study drugs will be provided by Shanghai Green Valley Pharmaceutical Co., Ltd. (Shanghai, China); however, they will not be involved in the design, patient recruitment, treatment, data collection and analysis, or any other procedures that would bias the study results.

A nitroglycerin tablet (0.5 mg per tablet, manufactured by Shanxi Yuanjingkangye Pharmaceutical Group Co., Ltd., Shanxi, China) is allowed if the patient has an angina attack, in which case the patients will be asked to record the administration times and the number of doses in detail, and the investigators will collect this information at the next follow-up visit. If the angina persists after three doses of nitroglycerin, the patient will be asked to go to the hospital for further treatment. If the patient has other underlying diseases, concomitant medications necessary to manage these disease are allowed and should be recorded in the medical records and CRFs. However, any other Chinese herbal medicines or Chinese patent medicines that have effects similar to Danshen are not allowed throughout the study period.

### Data management and monitoring

A CRF will serve as the repository for all results obtained in this trial. Source data will be traceable to the source documents (original records or certified copies). All data management procedures will be detailed in the trial-specific data management plan.

In addition, a data monitoring committee (DMC) will be established that will consist of an independent chair and two other independent members. The DMC will make sure that the protocol has been followed without deviation, including ensuring informed consent forms have been signed before inclusion, all inclusion and exclusion criteria are met, all primary and secondary variables and serious adverse events are well documented, and the source data and the CRFs are consistent.

### Statistical methods

The statistical analysis of this study will follow intention-to-treat principles. A blinded statistician will analyze the data using SAS version 9.1 software (SAS Institute Inc., Cary, NC, USA). Normally distributed data will be expressed as the mean ± SD. Numeric data will be expressed as percentages. *P* < 0.05 will be considered statistically significant. Repeated measures analysis of variance will be used for comparison of measurement data between the Danshen extract and control groups at baseline and 10 days and 70 days after treatment. Quantitative data will be compared using analysis of variance and *t* tests, whereas numeric data will be compared using the chi-square test.

### Research objectives

#### Primary objective

Serum levels of Lp-PLA_2_ will be assessed as baseline data as soon as the patients are included. After 10-day treatment during patients’ hospitalization, Lp-PLA_2_ levels will be assessed again on the day of discharge and on day 60 after discharge.

#### Secondary objectives

The secondary outcomes are as follows:

1. The proportion of patients in each treatment group who have clinically significant changes as defined by the angina frequency score on the Seattle Angina Questionnaire
2. Carotid arterial intima-media wall thickness
3. The frequency of angina attacks per week
4. Angina grade according to the Canadian Cardiovascular Society Angina Grading Scale
5. Consumption of short-acting nitrates
6. Changes in electrocardiogram results
7. Changes in serum lipid levels, high-sensitivity C-reactive protein levels, and the rate of platelet aggregation

These outcomes will be assessed at the same time points as the primary outcome. The safety of Danshen extract therapy will be evaluated by the incidence of new-onset major vascular events, overall mortality, and incidence of severe and moderate hemorrhages during patients’ hospitalization and 60 days after discharge. After each interim analysis, the DMC will determine whether the study can continue or there is a need to modify or terminate the study.

### Ethics

This trial has been approved by local institutional ethics committees of Taizhou Fourth People’s Hospital. It will be conducted according to the principles of the Declaration of Helsinki (Edinburgh 2000). Informed written consent forms will be signed by all participants before entering the trial.

## Discussion

Danshen, also known as *Salvia Militorrhizashen*, is considered one of the most important traditional Chinese medicines and has been widely used in Asian countries. It has been used for hundreds of years in the treatment of numerous ailments, including cardiovascular disease [14]. According to Chinese medicine theory, Danshen promotes blood flow and resolves blood stasis [25]. Although Danshen or its ingredients, including DanshenDuofensuanyan (salvianolate) and Danshen drop pills used in this study, improve the outcomes of patients with cardiovascular disease [26, 27], the mechanism of action remains unclear [28–30]. Inflammation plays an important role in the etiology of atherosclerosis and CAD [31]. We thus hypothesized that Danshen may improve the clinical outcomes of patients with CAD through its anti-inflammatory effect.

Lp-PLA_2_ belongs to the superfamily of PLA_2_ enzymes which are produced by macrophages that appear to play a role in the atherosclerotic vessel wall. Lp-PLA_2_ can be identified in the circulation and in atherosclerotic plaque and could act as an inflammatory marker with potential use as a predictor of cardiovascular risk and as a therapeutic target [32]. Emerging data suggest Lp-PLA_2_ is an independent predictor of risk [22] and may be superior to other inflammatory markers owing to its unique effects on the initiation and progression of atherosclerosis [33, 34] and minimal biovariation.

To our knowledge, our present study is the first double-blind, randomized trial to assess the effect of Danshen therapy on Lp-PLA_2_ level in patients with stable angina pectoris. The correlation between changing of Lp-PLA_2_ level and improvement of patient symptoms using other assessment tools, including the Seattle Angina Questionnaire, frequency of angina attacks, angina grade, consumption of short-acting nitrates, and so forth, will also be analyzed. It is well documented that a randomized controlled trial is the gold standard for evaluating the clinical efficacy and safety of a Chinese medicine and for providing critical evidence to develop and guide treatment strategies. Our results may elucidate the possible mechanism behind Danshen treatment effects in patients with cardiovascular disease and also may validate an objective blood test (LP-PLA_2_ level) for assessing the effectiveness of Danshen therapy in patients with stable angina pectoris.

However, our study still has limitations. First, because DanshenDuofensuanyan injection will be used only during patients’ hospitalization for 10 days and Danshen drop pills will be used during the 60 days after discharge, we cannot use blind the patients after discharge, given the need for Danshen drop pill prescription. Second, statins have been proven to decrease the LP-PLA_2_ level in previous studies. As such, although a placebo control will be applied in our study, we can assess only the complementary effect of Danshen, because statins are a standard of care treatment for patients with stable angina pectoris. Finally, given budget limitations, the follow-up time is not long enough to determine whether the change of Lp-PLA_2_ level can improve patients’ long-term cardiovascular outcomes.

### Trial status

Patient recruitment began on 5 September 2016. The trial is currently recruiting patients.

## Additional file

Additional file 1: SPIRIT 2013 checklist: The red text indicates on which page the corresponding content appears. P indicates page; NA indicates not available. (DOC 118 kb)

## Abbreviations

Blood RT: Blood routine test
CAD: Coronary artery disease
CRF: Case report form
DMC: Data Monitoring Committee
DOLPHIN study: Effect of Dan-shen Extract on Lipoprotein-associated PHospholipase A2 Levels in Patients with Stable Angina Pectoris study
Echo: Echocardiography
EKG: Electrocardiography
IMT: Intima-media thickness
Lp-PLA_2_: Lipoprotein-associated phospholipase A_2_
SPIRIT: Standard Protocol Items: Recommendations for Interventional Trials
